# An interdisciplinary educational path to understand the economic phenomena of a fluid and complex world with mathematics

**DOI:** 10.1007/s00500-023-08377-5

**Published:** 2023-05-23

**Authors:** Giovanna Bimonte, Francesco Saverio Tortoriello, Ilaria Veronesi

**Affiliations:** 1 Department of Economics and Statistics, University of Salerno, Fisciano, Italy; 2 Department of Mathematics, University of Salerno, Fisciano, Italy

## Abstract

In this paper, we describe an activity that involves economics and mathematics. It is included in the planning of orientation paths towards university studies within the Mathematical High School Project and is dedicated to students in the last years of high school. In particular, this research will deal with the issue of solving an economic problem using not only real analysis instruments but also geometrical topics concerning Euclidean geometry and topology. Mathematics becomes a language to understand and explain a real life problem, such as determining the optimal position of an airport, a nuclear reactor and so on. Some activities made use of dynamic geometry software and computer simulations.

## Background

In everyday life we often come across the need to express a decision, sometimes for personal reasons, other, more onerous, for professional, ethical or social ones. To make a reasoned choice, it is necessary to know the underlying dynamics in order to produce effective predictions (Morin 2001).

School is the context where the younger generations acquire knowledge, achieve skills and gain competences in the various disciplinary sectors. The main goal of education is to help students to build critical thinking and get social and ethical awareness that will guide them throughout their lives. Unlike what has come in the various centuries and until the last decades of the last century in which the school was confronted with a world whose development was constant but slow and therefore the school was able to remain the same for a long time, in the recent decades there has been an acceleration in changes in social and economic dynamics. Contemporary society is defined “fluid” precisely because of its rapidity in changing and is characterized by a very high degree of technology. It is the task of the school to identify the well-being of all people as a main topic and place it at the centre of social and economic dynamics. These dynamics have to be oriented in terms of economic stability and above all on the plan of psychological serenity (Baumann 2000). Sen defines capability as “a person’s ability to do valuable acts or reach valuable states of being; [it] represents the alternative combinations of things a person is able to do or be” (Sen 1993).

In higher education, an individual's capacities cannot be examined independently, as its conceptual framework presupposes a social construction of individuals interacting and competing, making individual and collective choices. Capabilities, of course, exist within and outside this framework, as they include both innate traits and skills acquired in a dynamic and fluid social environment.

Sen argues that capabilities should not only be seen as a means to a certain end, but rather as the very purpose of the educational process (Sen 1985; Saito 2003). Sen's (1985, 1993) capability approach challenges human capital theory, which sees education as an ordinary investment undertaken by individuals. He also remains sceptical of structuralist and post-structuralist approaches, which advocate the dominance of institutional settings and power over individual acts. Skills have both intrinsic and instrumental value. Material resources can be obtained through people's innate talents and acquired skills. The intrinsic value of resources is perceived by individuals as a means to a socially responsible end.

A complex reality requires a complex thinking capable of interpreting the dynamics of society’s changes in order to be able to make decisions that can be favourable for the private life but also for ethical or social professional choices whose fallout is much wider on the community. To make an informed and reasoned choice it is necessary to know all the dynamics in order to be able to produce effective forecasts (Morin 2001).

Education is not something removed from the cultural and social context, it exists and is structured within it. The school can develop methodologies capable of providing these tools so that the child, the adult of tomorrow, will be able to find his role in the multiple contexts and possibilities that life offers.

Therefore it is essential that the school, the training institution par excellence that helps future generations to plan their future, provides young people with tools and competences also in this sector. Unfortunately, in most curricula of the Italian school, also in the upper secondary one, the economy is the great absence in the face of the fact that about 30% of students enrol in University courses in economics.

Economics also shows a close interconnection with mathematics because it demonstrates how solving real life problems requires economic interpretations which are elaborated and built through a mathematical infrastructure. Suffice it to say that a large number of Nobel laureates in economics have been awarded to mathematicians. It should also be added that from the point of view of teaching, mathematics and economics constitute the bridge between the various disciplinary areas such as history, philosophy, literature, science and physics, and allow us to interpret historical and cultural moments from the past to our present in a unified vision.

On February 22, 2018, the document National Guidelines and New Scenarios was presented to MIUR that references to the 17 objectives of the 2030 agenda for sustainable development, in particular Objective 4 requires to "Provide quality, fair education and inclusive, and learning opportunities for all" and about mathematics it is written:

*Mathematics provides tools to investigate and explain many phenomena of the world around us, promoting a rational approach to the problems that we face in real life and therefore providing an important contribution to the construction of a conscious citizenship.*

*Mathematics (...) also allows you to develop important transversal skills through activities that enhance the typical processes of the discipline: "In particular, mathematics (...) contributes to developing the ability to communicate and discuss, to argue correctly, to understand the views and arguments of others. " These skills are relevant for the formation of an active and aware citizenship.*

and again:

*The mathematics laboratory represents a natural context to stimulate the ability to argue and stimulate peer comparison: (…) “In mathematics, as in other scientific disciplines, the laboratory is fundamental, intended both as a physical place and as a moment in which the pupil is active, formulates his own hypotheses and controls their consequences, designs and experiments, discusses and argues his choices, learns to collect data, negotiates and builds meanings, leads to temporary conclusions and new openings the construction of personal knowledge and collective. " In light of the description that is given in the Indications, the laboratory can also be a gym to learn how to make informed choices, to evaluate the consequences and therefore to take responsibility for them, aspects which are also central to education for an active and responsible citizenship.*

Starting from what emerges in the new scenarios regarding national indications, in the context of the activities of the “Mathematical High School Project'' promoted by the University of Salerno, researchers decided to introduce educational interdisciplinary modules on strongly impacting themes in the life of youngers. The “Mathematical High School Project'' is a didactic research project developed by the Mathematics Department of the University of Salerno with the collaboration of many departments of the same university. This project currently is extended nationwide and involves more than 160 institutes and 27 universities across the country. The idea of MHS is based on additional courses in extra-curricular hours mainly based on laboratory activities designed by working groups of researchers. In additional laboratory courses, mathematics intends to bring itself in a dialectical relationship with the other disciplines, as a cultural glue and a bridge between the two cultures, the humanistic and scientific one, into the curricular paths. This interdisciplinary mathematics is closely interconnected with the experiences of daily life, a mathematics that is not (for this reason) banal, on the contrary, which presents themes usually not addressed in regular school curricula and becomes a moment of reflection on daily reality. In addition to the relationships of mathematics with literature, history, philosophy, physics, chemistry and biology, topics related to the most advanced technologies and contemporary economic frameworks are analysed with the aim of demonstrating how mathematics is not only a key to reading but a key to resolving political and economic problems. The goal is rediscovering the role that mathematics has had over the centuries as a language and model of rational thought (Rogora and Tortoriello 2021) also with a view to verticalization of the curriculum for technology-assisted learning from high school to university. University students are offered educational training courses with new technologies (e.g. Bologna et al. 2020) in this vision of an educational unicum.

The recent developments in mathematics provide the potential for profound and highly beneficial changes in mathematics education at all levels, such as in economy. For example, the topic "game theory" or "decision theory" are subliminally present in everyday life and require mathematical skills to make advantageous choices. However, they are not included in the curricula of Italian high schools. This happens because in order to solve problems about these topics with the methodological approach used usually, students have to study differential and integral calculus with multiple variables, and calculate differential equations and solve even very complex systems. It is therefore unthinkable to present this theme to high school students since they do not have the prerequisites that allow them to understand and solve optimization problems.

In this work we present a didactic path developed in the last class of a scientific mathematical high school in which through participatory lessons, with cooperative learning modalities in group activities, the fundamental themes of game theory were presented to students.

## Decision theory and game theory

Decision Theory is the set of mathematical theories, logics and philosophies related to decision-making by rational individuals, i.e. individuals in competition or in groups. This theory includes utility theory, social choices and game theory. Decision theory has become an integral part of important approaches from the philosophy of science to ethics, passing through the theory of rationality.

A decision, made by an individual or a group, involves a choice between two or more actions, which determine different results. The applications of the theory range from abstract conjectures on ideally rational agents to practical suggestions for solving specific decision-making problems. Decision theorists investigate the logical consequences of different decision-making rules or explore the logical-mathematical aspects of different descriptions of rational behaviour; the applied ones are instead interested in examining the decision-making processes as they take place in reality.

From this point of view, it is usual to distinguish decision theory into two main strands: normative theory and descriptive theory. Those who deal with descriptive theory try to find out how decisions are taken in different operational contexts; those who deal with normative theory analyse the way decisions should be taken by referring to ideally rational agents.

Any decision, whether individual or group, involves a choice between several alternatives, or actions, or acts, each of which will produce one of several consequences that will depend on the conditions of the context, state of nature, in which the decision-making process takes place. Decisions, therefore, are made up of actions, states and consequences, with the latter depending, in most cases, on the action and the state in which the action takes place.

When analysing a decision problem, the analyst, who can be the same subject that takes the decision, must identify the relevant set of actions, states and consequences in order to characterize the problem itself in an adequate way. Through the identification of actions, states and consequences and the construction, if necessary, of a decision table or tree, the decision problem is specified.

Some interesting issues are related to the specification of a decision problem. The first concerns the appropriate description of the states of nature. Every decision problem implies consequences that the subject of the decision considers better than others, otherwise there would not be a problem of choice. In this context, the principle of dominance, which says to exclude all alternatives that entail worse consequences, whatever the state of nature, than some specific alternative, is particularly relevant. If there is one alternative that dominates all the others, the principle of dominance leads to choosing that alternative and the problem decision-making is solved optimally.

The fundamentals of modern decision theory, the value theory, can be found in the work of J. Von Neumann and Morgenstern (1947). The two authors show how, on the basis of certain postulates or axioms of rational behaviour of the person who has to make a decision, it is possible to introduce a real-value function called, according to the context in which one operates, value or utility, so that a decision based solely on this function is in fact reduced to a choice made following one's own scheme of preferences.

For a set of rational behaviour axioms, related to a certain agent, there is a function—and it can be determined—at real values perfectly equivalent to its preference scheme. In other words, if the operator's preference scheme satisfies a specific set of axioms, then there is a function of value or utility for the agent, and if the agent regulates his conduct based solely on value or utility he acts in accordance with his preference scheme.

In general, Decision theory investigates the rational behaviour of isolated individuals, Game theory deals with the rationality of the decisions of several people who depend on each other. The history of Game Theory is relatively short: it can be said that it was born in 1928, when John Von Neumann published his theorem of the MinMax, which allows us to find solutions for certain types of games. Von Neumann and Oskar Morgestern published the book "The Theory of Games and Economic Behaviour", and in 1950, John Forbes Nash published his doctoral thesis in which he demonstrated his famous theorem on Nash's Equilibrium. Game Theory is a branch of mathematics exploited in many fields: Economics, Politics, Medicine, Finance, Biology, Marketing, war situations; because of the human and social component that dominates the whole theory also scholars of Psychology, Sociology, Logic and Philosophy. Games can be represented in different forms: normal, extended and characteristic; the normal form foresees the use of tables, for the extended form the trees are used, the characteristic is the form used to represent cooperative games.

In economic analysis, Decision Theory is developed with a normative approach. More in general, Decision Theory is concerned with an agent’s choices, first defining a set of choices that a subject faces; a decision is the selection of one of these options. The decision analysis is focused on finding tools, methodologies, and software to help agents make better decisions. According to classical decision theory, due to her rationality an agent will decide to choose an action that has the highest subjective utility (Raiffa 1968). The recent developments in mathematics provide the potential for profound and highly beneficial changes in mathematics education at all levels.

Game Theory therefore acquires a significant value both in a positive key since it provides information to understand certain choices, strategies and tactics in conflict situations, and in a prescriptive key to determine when an equilibrium can occur and when not, in the interaction between two or more subjects. Given a problem solvable by mathematical programming, a model consists in representing the elements of the problem to be determined by decision variables, and the relations between them in the form of mathematical functions, one of which represents the value of the problem and is called objective function and the others which define which values of the variables are admissible and are called constraints.

Game theory aims to build mathematical models and find solutions, if any, from these models in relation to decision-making interactivity in which agents do not necessarily share the same interests.

## Case study: the game theory laboratory in the mathematical high school

### Contextualization

The project that will be presented in this article reports the experience of an activity carried out within the “Mathematical High School” Project in the school years 2020/21 and 2021/2022 with seven classes (11th and 12th grades) from different MHS in collaboration with Mathematics Department of the University of Salerno (Italy), Italy, 180 students were involved. The work is a collaboration with the research group of the Department of Mathematics with researchers of the Department of Economics of the University of Salerno.

The laboratory aims to provide mathematical and economic models that allow students to analyse and understand the dynamics of decisions in various contexts in order to build a unified vision of real historical-political-economic problems in an interdisciplinary perspective that goes beyond the traditional fragmentation of the school curriculum (Bimonte et al. 2020a, 2020b, 2021a, 2021b, 2021c, 2022).

Interdisciplinary topics are introduced to enrich students' curriculum by anchoring them strongly in the richness of emerging technologies, using their potential and designing technology-centred learning activities that are attractive to different spheres of knowledge (Tortoriello and Veronesi 2021).

The idea of the module is to involve students in active learning, exploiting their natural curiosity for economic and social issues, and to make them think about the questions before trying to give them answers based on theory (Bimonte et al. 2020a, 2020b, 2021a, 2021b, 2021c, 2022).

### The activities

Our topics reflect our purpose: in our laboratory we thought of activities that did not require advanced mathematical prerequisites and yet involved deep ideas of game theory and some refined mathematics.

Due to the Covid-19 pandemic the activities were all carried out in distance learning on e-learning platforms and consisted of 2 meetings of 2 and a half hours each, in extra-curricular hours in which the students worked both an entire class group on the platform and in the Breakout Rooms in subgroups that represented the noble Houses of the story in which the problems of the module were set.

We started proposing numerous examples to familiarize students with the problems of choice. We choose examples that can be categorized as "reality problems": the legislation of the ministry of education defines a “reality problem” as a problem that allows students to apply the acquired theoretical study to a specific case. Then we asked them to outline the strategies for obtaining a victory, also presenting the possibility that in the game may not "win" any player.

Some classic examples were presented to the students such as the *Beauty Contest*, *Tic-tac-Toe*, the *Prisoner's dilemma*, the *Hotelling linear market*, and more. We addressed these examples as open questions to be discussed in groups to propose possible solutions justifying the choices. The resolutive path to face was then rigorously and formally introduced, paying particular attention to the display form and the correct use of symbols and logical deduction rules.

Finally, the optimal solution, local and global, of the problem were presented, to make students understand how much mathematics permeates daily life, especially when referring to economic problems, choice problems or problems of maximizing or minimizing (depending on the case presented). To enable students to better understand the environmental impact of a human action, we asked them to give other examples in which a real problem must be answered taking into account numerous variables and conditions. The answers were full of ideas that highlighted the natural predisposition that the students have to face these issues.

Since the students did not know differential calculus, it was not possible to tackle optimal problems from the analytic point of view. We therefore chose to approach the topic using logical-deductive thinking or tools from a geometric and topological point of view. The main activity deals with the optimal positioning in the plane: teachers explained the tessellations of the plan, the Voronoi model and the triangulations of Delaunay. Thanks to the relevant observations through activities with special apps on the web and the repeated simulation of various examples with them, the use of ruler and compass first and then of dynamic geometry software, students were able to locate the optimal points in a plane that lead to the Nash equilibrium (Nash 1951). The activity will be described shortly in the next paragraph.

Specifically, the laboratory activities were constructed in such a way as to induce students to construct choice strategies, based on individual or group (collective) preferences, taking into account the more or less analytical knowledge required to solve the proposed games.

During the Lab, for example, we observed that in the game Tic-Tac-Toe all students use probability knowledge, not focusing on the structure of the game and its components, especially the existence of solutions.

The better known "Prisoner's Dilemma" always gets students with better skills a moment of comparison that easily brings them towards Nash equilibrium, even before the notion is formalized in the class.

Finally, optimal placement activities both on the straight line and in the plane involve several cognitive aspects mainly centred on geometry skills possessed by students that replace the lack of analytical knowledge needed to solve the problem formally.

Mathematical knowledge thus becomes the tool for constructing strategies for solving the proposed real problems, allowing us to observe how far the choices made deviate from the rational choices predicted by theory.

The students were then enunciated with the theorem which states that optimal points obtained thanks to the topology are exactly the Nash equilibrium points, demonstrating the generality of what they had achieved with skills acquired in previous years.

The lab activities guided students through a form of transition from a mathematical thinking in which concepts have an intuitive basis grounded in experience to one in which they are specified by formal definitions and their properties reconstructed through logical deductions.

### Methodologies

The activities of our path were developed with a constructivist approach (Harel and Papert 1991) where technological artefacts played the role of semiotic mediators (Rabardel 2002). It was chosen to develop the methodology of simulated role- playing: the students were divided into small groups and were given the role of landowners in medieval times with the task of protecting and prospering their lands by dealing with challenges, difficulties and the needs of the other territories. This student-centred approach, in a dynamic of challenge-situated learning, fostered active participation because it stimulated emotional intelligence. Subsequently, the theoretical contents were developed and treated in laboratory activities.

We observe that students usually spend little time planning solutions to proposed exercises and problems and are indolent about writing down the motivation behind their decisions.

This observation is highlighted by Schoenfeld (2016), who analysed the behaviour of university students when faced with a mathematical problem or exercise: considering numerical examples and thinking about their further generalization keeps solvers busy from the start and they quickly enjoy performing and writing something down, whereas discovering the immediate solution certainly requires some planning and writing.

This student-centred approach encourages active participation because it stimulates emotional intelligence (Goleman 1995) and relates everything to a context that specifies its meaning. Students, through storytelling, felt connected to the stories and placed themselves within the action; they faced real problems posed as a challenge or a difficulty with no immediate solution. This approach seeks to persuade the student to search for procedures and actions based on previous knowledge. The success and the failure in gaming actions allow students to construct new knowledge out of the experiences, learning from mistakes becomes the main approach to constructivist learning (Collins, 2008). The starting activity was the Beauty contest (Keynes 1936). It is an early theory of behavioural finance that describes how our perceptions of value can cause irrational fluctuations in presumably rational systems. Specifically, it describes how short-term fluctuations in the stock market are not caused by changes in the underlying value, but rather by investors' attempts to understand what others think the “average investor” finds of value. Trying to predict changes in the market or, more generally, the preferences of other players often turn out to be wrong. The problem posed is as follows: *Consider the numbers 1 to 100. Guess which number is closest to 2/3 of the average number declared by all players*.

The results, not surprising, tell us that in this guessing game the equilibrium implies that each player's belief is consistent with what all other players actually intend to choose. Actually, game-theoretic Nash equilibrium 0 is generally not reached if the game is one-shot, but is attained after a sufficient number of rounds in the iterative setting, i.e. in the presence of communication. Game theory predicts that if all players had identical beliefs and were perfectly rational, and if each player knew that all others were also perfectly rational, then 0 would be the unique and stable solution.

As the experiments show, most players do not behave this way. In fact, if only one player assumes that at least one player is irrational, then it is rational not to choose 0 as the proposed winning number.

It was interesting to note that once each player thought about the number he would declare, he was, in practice, already modifying the outcome of the game significantly. Even more surprising for the students was to discover that a rational player anticipating the argument of his opponents, also assumed to be rational, should have declared 1 as the best choice.

From the experimental data, it can be noted that the observed Winning Number is approximately the same in several experiments: a number between 30 and 40. The high variability of the chosen numbers reflects the players' uncertainty about the rationality of the others. The strategy chosen by an individual depends on his or her assumptions about the strategies of others, e.g. the distribution of strategies played at that time by others. All players have a minimal and context-dependent bias on the assumptions of others.

In our sample, consistent with what is predicted in the literature, the students chose a number in the range 30–40. We are surprised that a percentage of about 24% chose a number strictly greater than 67, which turns out to be impossible.

Tic-tac-toe is a game with a finite number of possible positions on the grid and a finite number of ways to fill the grid with different combinations of X's and O's.

An upper limit for the number of positions and the number of different games is given by 3^9^ = 19,683. This is the total number of possible game positions in a 3 × 3 grid, as each square will be an O, an X or a blank.

The game was presented in the extended form and the analysis of this game tree made it interesting to eliminate many possible positions as they are specular or rotated with respect to each other, focusing the analysis only on the distinct, isometric positions under reflection and rotation.

In the game Tic-Tac-Toe, it was interesting to note that the students immediately referred to the calculus of probability to find the solution to the game. We introduced the concept of Nash equilibrium in pure strategies, followed by that in mixed strategies. For finding equilibria in mixed strategies, after the existence theorem, we introduced the algorithm that consists of equalizing the payoffs of the two pure strategies of one player and finding, given a probability p, the mixed strategy of the other player that makes this equation true. Under the assumption that one player's mixed strategy must make the other player indifferent to the starting strategies, in order to solve the game and find equilibrium one must use one player's payoffs to solve the other player's strategy.

In that way, strategic interactions with other students/gamers imply the employment of constructing procedures to solve the assigned problem.

According to Schoenfeld and Sloane (2016), the cognitive mechanism of the phenomenon of the lack of a simple solution by individuals with mathematical knowledge is not well understood by the research community. In particular, they found that above-average university students often use sophisticated algebraic methods that lead nowhere rather than simple calculation methods that are within their reach. The explanation for this finding was given in the following terms: any problem evokes an image of a situation-problem that contains tentative solution beginnings, i.e. tentative general ideas to start the process of finding a solution. Students do not retain the knowledge most appropriate to the problem-situation because they have too few provisional solution beginnings in their possession.

In the Game Theory Lab we wanted to explore students’ attitudes between mathematical knowledge and strategic behaviours in solving non-routine mathematical problems.

Subsequently, the theoretical contents were developed and treated in laboratory activities where the teacher is not the holder of the knowledge to be transmitted: the collaborative and cooperative exchanges between students with the coordination of the teacher create the knowledge.

The plot of the activities has an important training potential, as it preserves and transmits practical knowledge capable of influencing human action and therefore represents a significant teaching–learning method that involves students in a total way and promotes a deeper knowledge of themselves and the world.

During the workshop activities, the teacher plays the role of "narrator of the history" within which the various questions of economics and mathematics have been built and proposes questions open to as many solutions as possible to the groups of students. Within the groups, through the dialectic of comparison, students examine the possible answers and elaborate their choice of resolution to the problems presented. Only then, at the end, the teacher collects the results of the online questionnaires and analyses the data by discussing them together with the students and comparing them with the results emerged in the historical experiments present in the literature. The role of the teacher is therefore an extremely dynamic role as he is the cultural mediator of information and knowledge that is oriented in the development of activities according to the students’ answers.

How not to give reason to Umberto Eco, when he said: "Reading stories means playing a game through which you learn to give meaning to the immensity of things that have happened and happen and will happen in the real world" (Eco 1994).

Bruner says: "Schools must cultivate their narrative ability, develop it, stop taking it for granted" (Bruner 1996).

## An example of an activity: optimal problem solved with topology

*“A finite set K* = *{1, ..., k} of retailers have located their facilities on S. A new retailer wants to maximize his marketshare after locating a new facility, depending only on the “distance” variable.”*

The activity started asking students to divide the space according to the rules mentioned above in the case of two facilities. Using first ruler and squares and then Geogebra software, students were guided in creating each retailer's attraction domain.

The “two points” cases (figures below) were quite easy to solve because students knew the definition of the axis of a segment in geometry and obtained the partition of the space by building the axis of the segment joining the two points that correspond to the two retailers. In the first example the evident symmetry of the figure has led to having equal areas and therefore equal payoffs. The second case was easy to build but led the students to observe the possibility that payoffs could change based on the allocation of retailers and this goes against a natural intuition of equal space division.
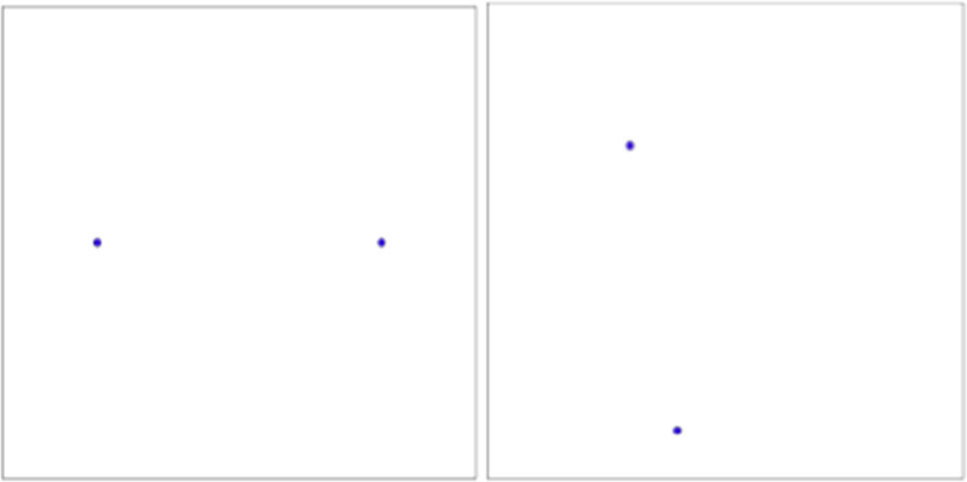


Even in the case of 3 points the students with ruler and squares were able to divide the space but the difficulty in developing the activity was already increasing.
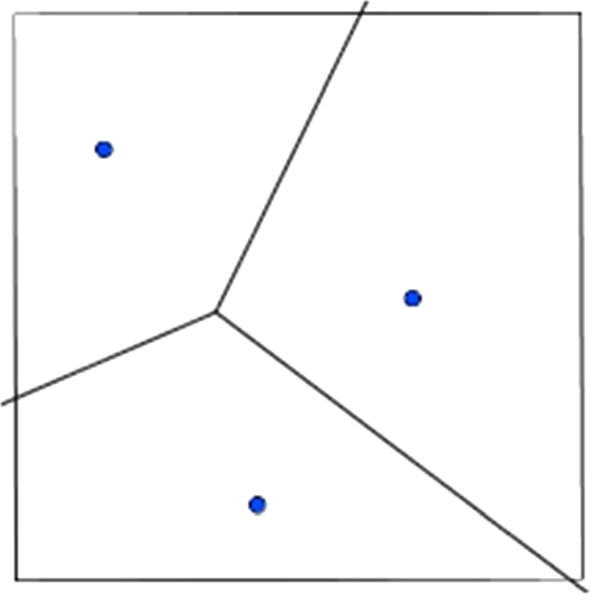


When the number of points becomes high it is convenient to introduce some geometric properties of the plane and some definitions. To trace the dominant regions of the players (i.e. their payoffs) in the intent of solving this locational optimization problem, we have to resort to Competitive Location Models. Without losing the rigour of the description of the procedure implemented, we will describe the essential steps regardless of the whole formal construct.

### Definition

*On the space S is defined a tessellation: Let*

$$ P_{{\text{K}}} : = \, \{ p_{1} , \, ..., \, p_{{\text{k}}} \} \subset S $$

*be a *finite* collection of points in S (where retailers have just opened a store),*

$V_{{\text{K}}} (p_{{\text{k}}} ) \, : = \, \{ y \in S \, : \, d \, (y, \, p_{{\text{k}}} ) \, \le \, d \, (y, \, p_{{\text{j}}} ){\text{ for all }}p_{{\text{j}}} \in P_{{\text{K}}} \}$ *is the Voronoi tessellation of S induced by P*_*K*_ *.*

The cell V_K_ (p_k_) contains all points whose distance from p_k_ is not larger than the distance from the other points in P_K_.

Call

$$ {\text{V }}\left( {P_{{\text{K}}} } \right) \, : = \, \left( {{\text{V}}_{K} \left( {p_{{\text{k}}} } \right)} \right)_{k \in K} $$

the set of all Voronoi cells V_K_ (*p*_k_).

We interpret λ(V_K_ (*p*_k_)) as the mass of consumers who are weakly closer to p_k_ than to any other point in P_K_. These consumers will weakly prefer to shop at location pk rather than at other locations in P_K_ since we assume that all retailers offer the same goods at the same price.

S is a compact subset of some Euclidean space, that λ is absolutely continuous with respect to the Lebesgue measure on this space and

$$ \lambda \left( {{\text{V}}_{{\text{K}}} \left( {p_{{\text{j}}} } \right)} \right) \, > \, 0{\text{ for all }}p_{{\text{j}}} \in P_{{\text{K}}} . $$

This assumption implies that the set of consumers that belong to r different Voronoi cells V_K_ (p_k1_), …, V_K_ (*p*_kr_) (i.e. are at the same distance of several points in *P*_K_) is of zero measure.

On *S* compact subset of *R*^2^ we define the Euclidean distance function $\|\cdot\|$. The dominance region is called the “ordinary Voronoi polygon” associated with p_k_, and the partition V_K_ (P_K_) is the planar ordinary Voronoi diagram generated by P_K_. The edges of Voronoi polygons in *R*^2^ are line segments.

For each retailer, action will be identified with a position (a point) in the plane.

The payoff of player *i* is

$$ {\text{f}}_{{\text{i}}} \left( a \right) = \sum_{{{\text{J}} \in {\text{K}}}} \lambda \left( {{\text{V}}_{{\text{J}}} \left( {a_{{\text{i}}} } \right)} \right) $$

That is the measure of the consumers that are closer to the location that they choose than to any other location chosen by any other player.

**Fig. 1:**
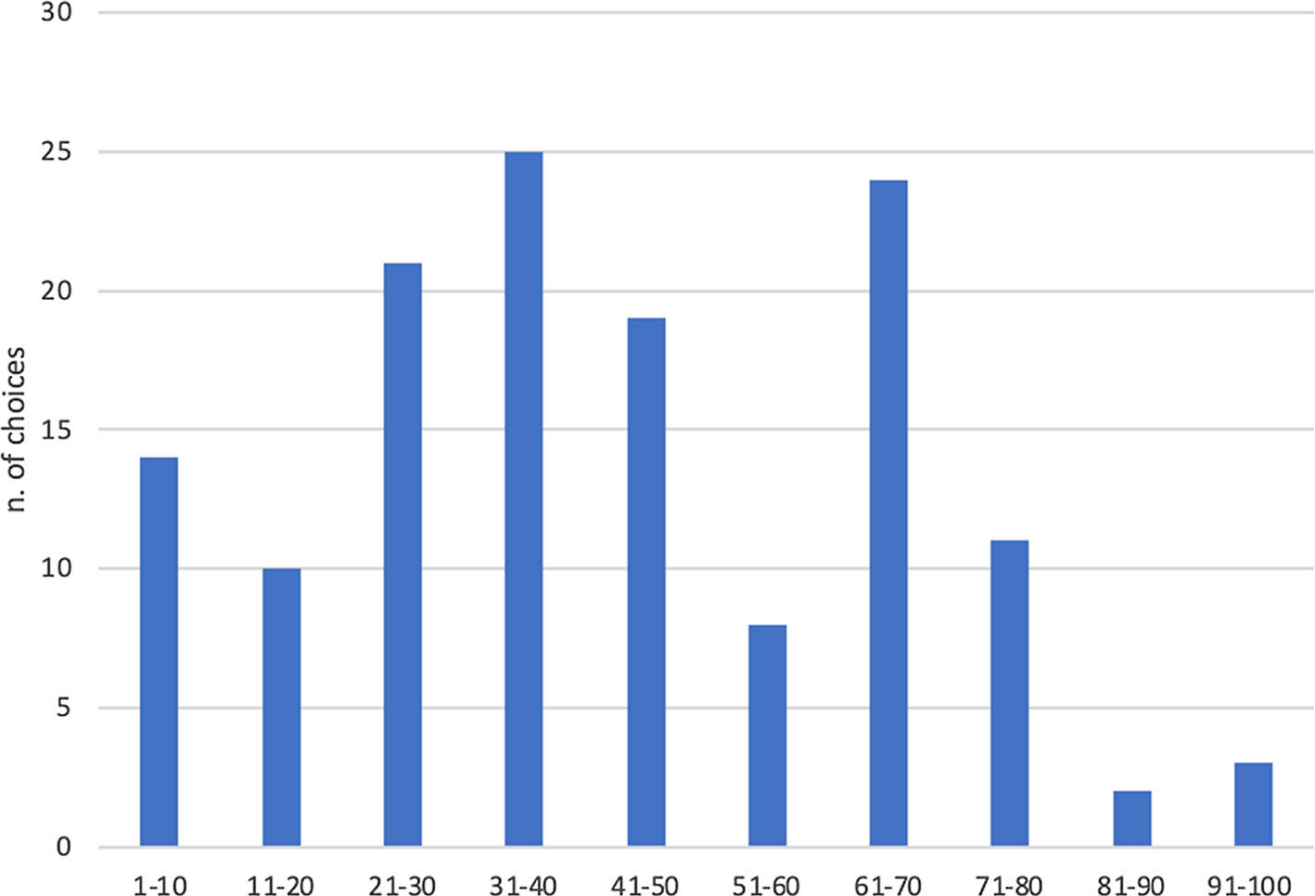
The Beauty Contest’s answers

**Fig. 2:**
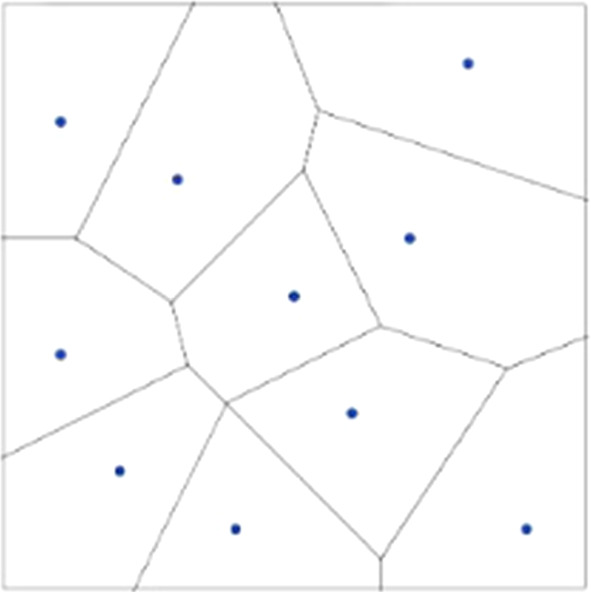
The payoff of players (Voronoi tessellation)

**Fig. 3:**
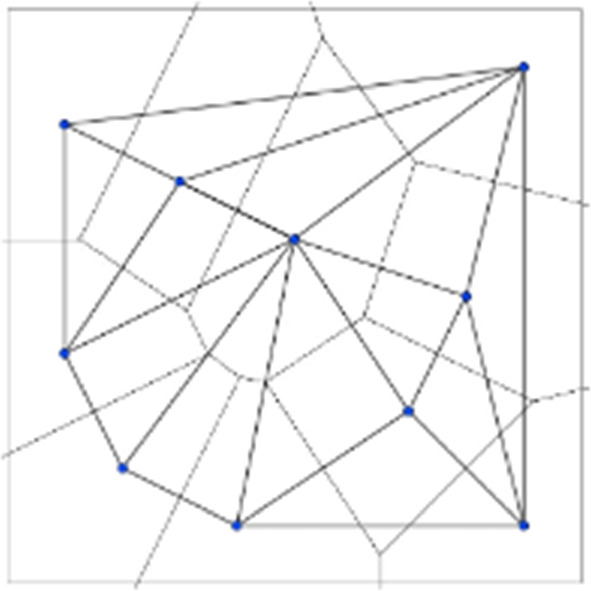
The Delaunay triangulation

**Fig. 4:**
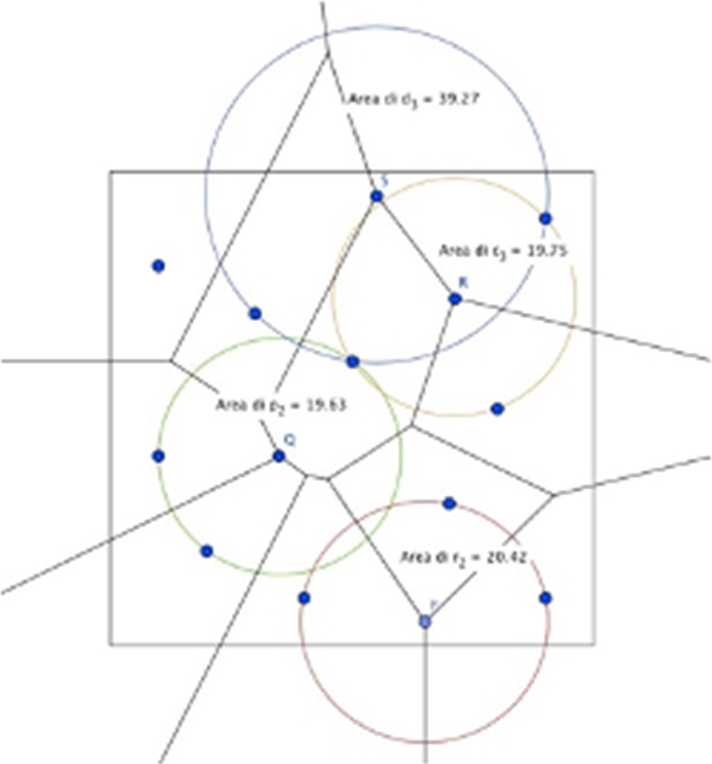
The circumferences circumscribed to the Delaunay’s triangles

**Fig. 5:**
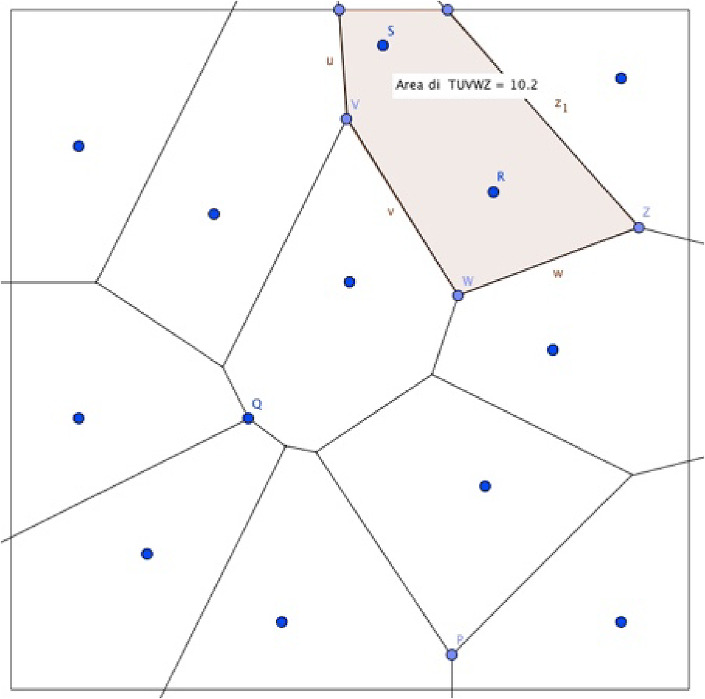
The new configuration, under Voronoi solution, with the location of the new retailer with the maximum area

Under these assumptions, the location of the new facility is determined by the maximization of the distance from other existing facilities: i.e. the retailer decides to locate the new store in the farthest point with respect to all existing stores. The task of determining this location is the largest empty circle problem (Figs. 1, 2, 3, 4, 5).

### Definition

*The Delaunay Triangulation, *DT* (P*_K_*), for a set of points P*_K_* is a triangulation such that no point in P*_*K*_* is inside the circumcircle of any triangle in DT (P*_K_*). The edges of DT (S) are called Delaunay edges.*

### Lemma 1

*Any two points of S are joined by a Voronoi edge if and only if their Voronoi regions are edge-adjacent.*

Students, thanks to the software Geogebra, drew the Delaunay triangulation and the circumferences circumscribed to the triangles, then they determined the circle of maximum area.

They verified that if a circle had the greatest area but part of it was external to the plane S (object of the search for the point of maximum gain), the area included in S was in any case smaller than the largest empty circle entirely contained in the space S.

The largest empty circle has these characteristics:

- its centre is in the interior of *S*,
- the circle contains no points from *P*_K_ inside it,
- it is the largest in the sense that there is no other circle with strictly wider area contained in *S*,
- it is fully included in S.

Students used the Voronoi diagram of *P*_*K*_ to solve this problem and find the location of the new facility adding to the points already known also R, the centre of the widest circle.

It can be shown that if the centre of a largest empty circle is strictly inside the convex hull of *P*_*K*_, then the centre coincides with a Voronoi vertex.

After completing the geometric construction and solving the search for where to allocate the new facility as a function of distance only, it has been explained to students that the Voronoi problem can be approximated by an isolation game with a measure of distance and that the solutions of the isolation game correspond to the Nash equilibrium points obtained by solving the maximum distance problem analytically in multivariable functions, a topic that would not have been possible to deal with in a high school since there are no mathematical prerequisites necessary for students. Then the configuration of the isolation game model was rigorously defined to students describing that players’ strategy set in the isolation game is the entire S.

## Conclusion

The activity presented to the students starts with the guided construction of a game and a discussion on the existence of possible solutions. Many solution concepts were analysed and addressed for different games. The main objectives of the workshop were to determine the students' ability to reflect on real life problems from another point of view. To achieve this goal, the students were led to decrease the value of probability to solve strategic situations.

Students were led to use mathematical thinking to promote the idea of trade-offs between social and individual choices when faced with different life scenarios. They assessed the difficulty posed by different rationality choices to advance strategic thinking on the analysis of critical factors and variables that will influence a decision, taking into account that other players will act in the same way.

The laboratory activities guided the students through a form of transition from mathematical thinking in which concepts have an intuitive basis based on experience to one in which they are specified by formal definitions and their properties reconstructed through logical deductions.

We divided the students into small groups that could coordinate within a virtual classroom (breakout rooms). As we had foreseen in the strategic choice to develop the activities with the methodology of simulated role-playing, a natural competition arose among the various groups, which made the activities much livelier and more participative.

The first problems were easy in order to become familiar with more complex problems. When the students faced the *Election Game*, they expected a correct answer, a solution to the game about choosing a knight to lead the army. In general, students are familiar with mathematical problems and their solutions. It was surprising to be confronted with a problem that could not be solved in one way or, worse, could have no solution at all. On the other hand, for the game of *Tic-tac-toe*, they were sure of the solution in mixed strategies: the concept of probability comes naturally to the students, perhaps because it is a well-known game. Once again, prior knowledge comes to the aid of finding the solutions and equilibrium of a game or finding the optimal choice.

Finally, in the Battle of sex they did not accept that a game could have two solutions (Nash equilibrium) and that they had to define another selection criterion between the two.

When students tackled the positional problem, many of them identified the best regions, aided by visual skills. In the linear city (Hotelling), most students identified the centre as the best solution in terms of maximizing each player's area (city portion). Some students had a physical problem approach, as if the agents were stationary and could no longer change their minds: the reasoning involved is never dynamic. They are generally not used to thinking about all the possible consequences of their own and their opponent's actions.

When the students faced the problem in the plan, they recognized that the focus was on understanding the criterion behind the choice and tended not to justify the choice by saying that it was obvious, as in the linear case. When we put them in the plane, they used their knowledge of geometry, but we showed that this was no longer sufficient.

We have observed that in the development of laboratory activities, students tend to reserve little time for the strategic planning of actions and solutions and are not used to and encouraged to write the reasons for their decisions as they are aware of the need for formal rigour in justifying the choice of an action or operation.

On the other hand, a strategic interaction focuses on problem-based learning, where immediate feedback is provided: a classroom Game Theory Lab works on the principle of possible failure and often includes stories in which the student can participate.

Game theory applied in classroom laboratories includes good learning principles such as constructivism, experiential learning, situated learning and problem-based learning, direct instruction. When students work on a mathematical problem and find themselves trapped in a long chain of calculations, they cannot get immediate feedback and cannot learn from their mistakes, because they cannot spot them. Every mathematics problem, therefore, needs to be carefully designed so that it relates to real-world problems.

Students can learn the concepts and principles of mathematics insight Game Theory through the experience of finding various possible solutions to a complex problem (experiential learning). Teachers need to act as facilitators to guide students in developing critical thinking skills, stimulating the construction of knowledge and reflection on the learning process (constructivism).

Mathematical Game Theory has the task of fostering the understanding of domain-specific knowledge and thinking strategies in a coherent way, by placing rationality at the basis of choices, or questioning it.

## Data Availability

Enquiries about data availability should be directed to the authors.

## Declarations

### Human and animals rights

This article does not contain any studies with animals performed by any of the authors.
